# Factors influencing the use of health services by trauma patients according to insurance type and injury severity score in South Korea: Based on Andersen’s behavioral model

**DOI:** 10.1371/journal.pone.0238258

**Published:** 2020-08-27

**Authors:** Hyunju Kim, Younkyoung Kim

**Affiliations:** 1 General Surgery Department, Chonnam National University Hospital, Gwangju City, Republic of Korea; 2 College of Nursing, Chonnam National University, Gwangju City, Republic of Korea; University Hospital Zurich, SWITZERLAND

## Abstract

This study aims to understand the difference in trauma patients’ use of health services in Korea according to insurance type and the Injury Severity Score. Andersen’s behavioral model of health service use is employed to identify the factors influencing their use. Claims data from January 1 to December 31, 2016 were extracted from both the Health Insurance Review and Assessment Service and the automobile insurance screening center for all the medical treatments identified with the Korean Triage and Acuity Scale and Injury Severity Score. Using the Health Insurance Review and Assessment Service’s remote statistical analysis system, hierarchical regression and negative binomial analyses were conducted to determine the effect of predisposing, enabling, and need factors on health service use. The results demonstrate that the use of Korean health services is relatively equitable since medical expenses for trauma patients are greatly influenced by need factors. However, the length of time trauma patients stay in the hospital appears to differ according to insurance type. This study suggests that healthcare policies need to increase coverage benefits and improve medical billing for patients with severe trauma, as well as develop a more robust screening system for patients with mild to moderate impairments.

## Introduction

The mortality rate per 100,000 population (standardized rates) in Korea from trauma (e.g., transport accidents, falls, drowning, burns, and poisoning) continued to be higher from 2000–2016 compared to the rates in other Organization for Economic Co-operation and Development (OECD) countries, such as the United Kingdom, Canada, Germany, and Japan [1]. Consequences of traumatic accidents, which occur relatively frequently in socially and economically active young and middle-aged adults, are not only a loss to the nation but also increase individual expenditure due to direct financial loss and accident-induced disabilities [2]. The socioeconomic burden from trauma is greater than that from all other health problems, and socioeconomically disadvantaged people are more vulnerable to trauma [3]. This can intensify the health disparity and social inequality between income classes. Hence, providing appropriate healthcare service to resolve trauma-induced health problems early on and minimize trauma-induced disabilities is critical for the nation.

Insurance type is known as an important determinant of inequality in healthcare utilization for disadvantaged individuals such as medical aid recipients and the low-income class [4]. With workers’ compensation excluded, the general insurance for Korean trauma patients today can be broadly divided into national health insurance (NHI), medical aid, and automobile insurance [5]. Korea has implemented the national health insurance system (NHIS) as a form of social insurance since 1989. The Korean government provides medical aid for the low-income class because of their limited access to healthcare due to financial reasons. Several conditions, including basic income levels, are considered, and all Koreans are covered by either NHI or medical aid. Unlike NHI and medical aid, which are provided to all Koreans through the government, automobile insurance is a private insurance service that automobile owners must sign up for to recover damages caused by car accidents. However, previous studies have indicated that medical aid beneficiaries have long hospital stays, but insufficient medical capacity [6], and automobile insurance is a matter of unnecessary hospitalization and excessive medical treatment [7]. Studies have attempted to examine the differences in healthcare utilization by trauma patients according to insurance type. However, they only examined healthcare utilization in patients with automobile insurance claims [7,8], which limited their investigation of healthcare utilization among trauma patients to the three types of health insurance.

To compare healthcare utilization by insurance type, ensuring homogeneity of various conditions among the participants is important. Specifically, injury severity must be accommodated to ensure consistent conditions for healthcare service needs among trauma patients [9]. The injury severity score (ISS) is a scoring system developed to assess the severity of an injury in traffic accident victims, with higher scores indicating greater severity of injury. The fatality rate was close to 30% in the severe injury group, and the fatality rate increased with increasing ISS score [10]. Apart from its use in assessing injury severity in trauma patients, the ISS is an anatomical scoring system effective in predicting mortality, hospital stay, length of recovery, and demands for resources to manage trauma patients [10]. Moreover, it is a major predictor of healthcare utilization in such patients [11–13] and is actively utilized in healthcare providers’ communications and triage [9]. Therefore, analyzing healthcare utilization in trauma patients according to the ISS is a systematic method of analysis, where trauma patients’ healthcare service needs are relatively homogeneously divided.

Among various models for healthcare utilization, Andersen’s behavioral model for healthcare utilization (hereafter, the Andersen model) has been validated for factor classification by multiple studies. It has also been recognized to comprehensively account for individuals’ internal and external factors, and to shed light on the relative gravity of different predictors of healthcare utilization [14]. This model explains how healthcare utilization is influenced by three factors: predisposing, enabling, and need factors. Predisposing factors contribute to one’s health status and healthcare utilization, namely sociodemographic factors [15], while enabling factors describe the variation in available resources for individuals that may impact their healthcare utilization [16]. Need factors encompass individual’s perceived or clinically assessed needs for health, functional capacity, physical signs, and general health status [16]. Studies that applied the Andersen model found that need factors are strongly associated with the number of emergency department (ED) visits in individuals with substance-related disorders [17]. Further, a study that examined the equity in initial access to formal dementia care in Europe reported that predisposing factors such as area of residence, sex, and age are important factors for determining access to care [18]. Therefore, examining trauma patients’ healthcare utilization based on a theoretical framework like the Andersen model would enable a more systematic and comprehensive explanation.

This study aims to compare and analyze trauma patients’ healthcare utilization by insurance type and the ISS, and identify its predictors based on the Andersen model using healthcare big data supplied by Korea’s Health Insurance Review and Assessment Service (HIRA) [19]. By examining the equity in healthcare utilization among trauma patients by insurance type through the classification of their medical needs and predicting their healthcare utilization patterns, we will present evidence for healthcare policymaking and policy changes. This will ultimately shed light on the direction of care to promote early recovery with minimal complications in severe trauma patients by helping them properly utilize healthcare.

## Materials and methods

### Design

This study is a retrospective, descriptive survey aiming to compare and analyze trauma patients’ healthcare utilization by insurance type and the ISS, and identify its predictors based on the Andersen model.

### Participants

Participants were selected from claims sent to the HIRA with the Korean Triage and Acuity Scale (KTAS) and the ISS, specified from January 1 to December 31, 2016. We requested all data for care given on or before June 30, 2017. Colombo et al. [20] defined the period of treatment from onset to six months as the subacute. Therefore, we included inpatient and outpatient care records from onset to six months to analyze trauma patients’ healthcare utilization during the acute treatment phase. Since the Korean government reorganized the system to apply KTAS and ISS at level I medical institutions in 2016, the patients who were assessed KTAS and ISS are treated at level I medical institutions (the regional trauma center). Deaths within 48 hours after trauma were excluded from the analysis, considering that deaths within 48 hours have a large variation in the use of medical services [21].

A total of 72,380 NHI *bills*, 5,733 medical aid *bills*, and 36,272 automobile insurance *bills* were obtained. We selected 7,514 NHI and medical aid recipients having KTAS and ISS among them and excluded 2,448 patients whose trauma occurred after December 31, 2016. From the remaining 5,066 patients, 12 were excluded as they received care for more than six months, beyond the acute phase. This yielded a total of 7,334 participants for analysis: 4,733 with NHI, 321 medical aid recipients, and 2,280 automobile insurance subscribers (S1 Appendix).

Per the revised emergency medical fee schedule, the HIRA requires regional level I trauma centers to specify KTAS and ISS on their claims for care provided on or after January 1, 2016. The KTAS is a triage system for patients admitted to an ED to enable prompt treatment for patients with severe conditions. This system requires immediate treatment for patients at level 1, where immediate resuscitation is required. Level 2 patients (emergency) are required to receive treatment within 15 minutes, level 3 patients (urgent) within 30 minutes, level 4 patients (less urgent) within one hour, and level 5 patients (non-urgent) within two hours [22].

### Measurement variables

#### Predisposing factors

1. Sex: classified into male and female as specified on the claim.
2. Age: determined based on the age in full years specified on the claim.
3. Date of onset: set as the first day of care and classified by season: spring (March–May), summer (June–August), fall (September–November), and winter (December–February).

#### Enabling factors

1. Duration of ED stay: specified for code MS005 (CCYYMMDDHHMM/ CCYYMMDDHHMM) and converted to minutes.
2. Insurance type: classified into NHI, medical aid, and automobile insurance as specified on the claims.

#### Need factor

1. Korean Triage and Acuity Scale (KTAS): Patients are classified into five levels (level 1–5), with level 1 indicating the highest emergency, requiring priority treatment.
2. Injury Severity Score (ISS): The score ranges from 1–75. In our study, scores were classified as mild (1–8), moderate (9–15), severe (16–24), very severe (25–40), and critically severe (41–75) [23].
3. Injury code numbers: indicate injury (S00–S99, T00–T14) per the injury code criteria by the HIRA.

#### Healthcare utilization

Healthcare utilization was defined as the total cost of care and days of hospital stay for six months from the onset of trauma. The claims with codes S00–S99, T00–T14 in the first, second, and third diagnosis per the HIRA injury standard criteria [24] were used.

1. Total cost of care: the sum of the cost of inpatient and outpatient care for six months after trauma.
2. Length of hospital stay in days: duration of days in hospital for six months after trauma.

### Data collection and procedure

Korea’s healthcare big data is created based on the claims for the cost of care covered by the NHIS made by each healthcare provider to the HIRA. NHIS claims data is an excellent dataset, representative of health services provided to the Korean public and reflecting the actual healthcare environment; using such big data would contribute to the evidence presented for healthcare policies [25]. We used a remote analysis system to analyze the healthcare big data supplied by the HIRA. The data is not disclosed to the public due to ethical and legal limitations but can be extracted and used by request through the submission of relevant documents to HIRA. The export of data itself is prohibited and only analyzed results can be exported (S2 Appendix).

We extracted NHI and medical aid data from the HIRA healthcare big data and extracted automobile insurance data from the Automobile Insurance Review Center. To classify NHI, medical aid, and automobile insurance groups based on similar characteristics, we studied trauma patients with the ISS and KTAS specified on the claims for care provided in 2016 to compare trauma patients’ healthcare utilization by insurance type. We excluded workers’ compensation patients as these claims are not addressed by the HIRA.

### Ethical considerations

As a measure to protect our study participants, we obtained approval for this study from the Institutional Review Board (IRB) at Chonnam National University Hospital upon review of the study purpose, inclusion and exclusion criteria, methods, data management and confidentiality, study information sheet, and participant consent (IRB No: CNUH-EXP-2017-152). All data were given an identification number instead of names for anonymity purposes and were stored in a password-protected file and locked cabinet only accessible by the researcher. We requested data storage in the HIRA server via an official letter; the data were deleted six months later.

### Data analysis

We utilized the SAS Enterprise Guide version 6.1 (SAS Institute Inc., Cary, North Carolina, USA) of the HIRA remote analysis system.

1. Participants’ demographic characteristics were analyzed with descriptive statistics.
2. Variation of healthcare utilization by NHI type and the ISS category was analyzed with ANOVA. Data that fulfilled the assumption of homogeneity of variance were tested with the Bonferroni post-hoc test, and those that did not were analyzed with the Welch test and the Dunnett post-hoc test.
3. The predictors of total cost of care were analyzed with hierarchical regression analysis. We first entered participants’ predisposing factors in the model, followed by their enabling and need factors, which were the focus of our study (insurance type and injury severity). Total cost of care data analyzed with multiple linear regression were log-transformed for the assumption of normality.
4. The predictors of length of hospital stay in days, a count data, were analyzed with negative binomial analysis.
5. The significance level was set to 0.05.

## Results

### Demographic characteristics

Participants’ demographic characteristics according to insurance type were as follows (Table 1). Men accounted for 69.7% of claims, more than twice as many as women, and were similarly distributed by insurance type. In terms of age, NHI and medical aid were the highest among those in their 50s at 19.0% and 28.7%, respectively, and automobile insurance was the highest for those in their 60s (18.2%). Trauma occurred most frequently in winter among the four seasons, followed by fall, summer, and spring. Emergency department stay was 262.09 minutes on average, with the shortest durations for automobile insurance recipients, followed by NHI and medical aid recipients. Overall, patients with KTAS level 3 or higher accounted for 79.6%, and patients with ISS with a score of 16 or higher were more than 40%. However, of the patients with ISS 1~8 indication (mild injury), those with NHI and medical aid had the highest rates of 39.7% and 33.0%, respectively. The average number of diagnosed injuries was 8.11, and deaths within 6 months of trauma were 5.6%. Total medical expenses were highest in medical aid, followed by automobile insurance and NHI. The average length of stay was 49.57 days, the longest for automobile insurance, followed by medical aid and NHI.

**Table 1:** General characteristics of the participants (*N* = 7,334).

Characteristics	Class	NHI n (%)	Medical aid n (%)	Automobile n (%)	Total n (%)
**Sex**	Male	3385(71.5)	219(68.2)	1,508(66.1)	5,112(69.7)
	Female	1,348(28.5)	102(31.8)	772(33.9)	2,222(30.3)
**Age (yrs)**	0~9	249(5.3)	7(2.2)	69(3.0)	325(4.4)
	10~19	247(5.2)	11(3.4)	126(5.5)	384(5.2)
	20~29	403(8.5)	15(4.7)	224(9.8)	642(8.8)
	30~39	379(8.0)	15(4.7)	223(9.8)	617(8.4)
	40~49	567(12.0)	32(9.9)	314(13.8)	913(12.5)
	50~59	902(19.0)	92(28.7)	393(17.2)	1,387(18.9)
	60~69	781(16.5)	57(17.7)	414(18.2)	1,252(17.1)
	70~79	719(15.2)	58(18.1)	371(16.3)	1,148(15.6)
	≥80	486(10.3)	34(10.6)	146(6.4)	666(9.1)
**Season of trauma**	Spring	857(18.1)	62(19.3)	374(16.4)	1,293(17.6)
	Summer	1,243(26.3)	78(24.3)	561(24.6)	1,882(25.7)
	Fall	1,306(27.6)	87(27.1)	660(29.0)	2,053(28.0)
	Winter	1,327(28.0)	94(29.3)	685(30.0)	2,106(28.7)
**ED stay (min)**^†^		266.98±265.58	306.51±351.53	245.80±267.25	262.09±270.71
**KTAS**	1 Resuscitation	383(8.1)	38(11.8)	333(14.6)	754(10.3)
	2 Emergency	1,861(39.3)	135(42.1)	1,227(53.8)	3,223(43.9)
	3 Urgency	1,288(27.2)	73(22.7)	502(22.1)	1,863(25.4)
	4 Less Urgency	1,069(22.6)	65(20.3)	100(8.3)	1,324(18.1)
	5 Nonurgency	132(2.8)	10(3.1)	28(1.2)	170(2.3)
**ISS**	Mild (1~8)	1,880(39.7)	106(33.0)	575(25.2)	2,561(34.9)
	Moderate (9~15)	1,012(21.4)	80(24.9)	514(22.6)	1,606(21.9)
	Severe (16~24)	1,030(21.7)	74(23.0)	650(28.5)	1,754(23.9)
	Very severe (25~40)	732(15.5)	57(17.8)	454(19.9)	1,243(17.0)
	Critically severe (41~75)	79(1.7)	4(1.3)	87(3.8)	170(2.3)
**Number of diagnosed injuries**		7.08±8.85	6.97±12.33	10.40±8.95	8.11±9.19
**Survival**	Alive	4,541(95.9)	308(95.9)	2,076(91.1)	6,925(94.4)
	Death	192(4.1)	13(4.1)	204(8.9)	409(5.6)
**Total medical expense (1,000 won)**		12,390±16.37	146,27±15,70	13,599±16,56	12,864±16,41
**Length of stay (days)**		28.61±38.93	47.28±53.39	55.20±48.93	49.57±53.96

^†^ED stay: Emergency department stay, including missing data (non-missing = 7286); NHI: National Health Insurance; KTAS: Korean Triage and Acuity Scale; ISS: Injury Severity Score

### Healthcare utilization according to insurance type and ISS

Variations in healthcare utilization according to insurance type and the ISS were as follows (Table 2). Differences in total cost of care were statistically significant according to insurance type (F (2, 7221) = 55.80, *p* < .001) and the ISS (F (5, 7329) = 534.54, *p* < .001). Total cost of care was higher in medical aid and automobile insurance claims compared to NHI claims and was lower with decreasing ISS; on the other hand, the very severe group (25–40) and critically severe group (41–75) had the highest total cost of care. Number of days of hospital stay was the highest with automobile insurance claims, followed by medical aid and NHI claims (F (2, 7331) = 212.62, *p* < .001). Number of days of hospital stay decreased with decreasing ISS (F (5, 7329) = 321.24, *p* < .001), but there were no significant differences according to the ISS in groups with severe, very severe, and critically severe injury (16–75).

**Table 2:** Health service use according to insurance type and ISS (*N* = 7,334).

Variable	Class		Mean	SD	F	*p*	Post-hoc
Total medical expense	Insurance Type	NHI^a^	15.70	1.16	55.80	<.001	a<b,c^†^
		Medical Aid^b^	15.98	1.11			
		Automobile^c^	16.02	1.19			
	ISS	1~8^a^	15.10	0.90	534.54	<.001	a<b<c<d,e^††^
		9~15^b^	15.81	1.00			
		16~24^c^	16.25	1.10			
		25~40^d^	16.52	1.16			
		41~75^e^	16.64	1.43			
Length of stay	Insurance Type	NHI^a^	39.81	48.67	212.62	<.001	a<b<c^††^
		Medical Aid^b^	60.03	59.77			
		Automobile^c^	68.36	58.15			
	ISS	1~8^a^	24.94	32.98	321.24	<.001	a<b<c,d,e^††^
		9~15^b^	54.67	52.58			
		16~24^c^	65.68	57.28			
		25~40^d^	67.38	63.37			
		41~75^e^	68.59	70.34			

^†^Bonferroni post-hoc test;

^††^Dunnett post-hoc test; Total medical expense: converted to log value and analyzed; NHI = National Health Insurance; ISS = Injury Severity Score

### Healthcare utilization by insurance type according to ISS

Variations in healthcare utilization by insurance type according to the ISS were as follows (Table 3). Among the patients with mild injury (ISS 1–8), total cost of care was greater with medical aid claims compared to NHI and automobile insurance claims (F (2, 2558) = 29.26, *p* < .001). Days in hospital care were greatest with automobile insurance, followed by medical aid and NHI (F (2, 2558) = 74.99, *p* < .001). Among patients with moderate injury (ISS 9–15), total cost of care was the highest with medical aid claims, followed by automobile insurance and NHI (F (2, 1603) = 10.43, *p* < .001). Days in hospital care were higher with medical aid and automobile insurance claims than NHI (F (2, 1603) = 40.79, *p* < .001). Among the patients with severe injury (ISS 16–24), total cost of care did not differ according to insurance type (F (2, 1751) = 0.51, *p* = .600). Days in hospital care were higher with medical aid and automobile insurance than with NHI claims (F (2, 1751) = 55.90, *p* < .001). Among the patients with very severe injury (ISS 25–40), total cost of care did not differ according to insurance type (F (2, 1240) = 1.01, *p* = .365). Days in hospital care were higher with medical aid and automobile insurance than with NHI claims (F (2, 1240) = 8.89, *p* < .001). Among the patients with critically severe injury (ISS 41–75), total cost of care (F (2, 167) = 1.03, *p* = .360) and days in hospital care (F (2, 167) = 1.11, *p* = .330) did not differ according to insurance type.

**Table 3:** Health service use by insurance type according to ISS.

ISS	Variable	Class	Mean	SD	F	*p*	Post-hoc
Mild (1~8) (n = 2,561)	Total medical expenses	NHI^a^	15.02	0.85	29.26	<.001	a,c<b^††^
		Medical Aid^b^	15.40	0.88			
		Automobile^c^	15.36	1.01			
	Length of stay	NHI^a^	19.34	26.60	74.99	<.001	a<b<c^††^
		Medical Aid^b^	33.64	44.15			
		Automobile^c^	41.62	42.21			
Moderate (9~15) (n = 1,606)	Total medical expenses	NHI^a^	15.73	1.01	10.43	<.001	a<c<b^†^
		Medical Aid^b^	16.05	0.91			
		Automobile^c^	15.95	0.96			
	Length of stay	NHI^a^	45.85	50.61	40.79	<.001	a<b,c^†^
		Medical Aid^b^	65.36	59.19			
		Automobile^c^	70.39	51.43			
Severe (16~24) (n = 1,754)	Total medical expenses	NHI	16.23	1.06	0.51	.600	
		Medical Aid	16.24	1.25			
		Automobile	16.29	1.15			
	Length of stay	NHI^a^	53.74	52.57	55.90	<.001	a<b,c^††^
		Medical Aid^b^	75.20	60.58			
		Automobile^c^	83.50	59.25			
Very severe 25~40 (N = 1,243)	Total medical expenses	NHI	16.55	1.13	1.01	.365	
		Medical Aid	16.66	1.04			
		Automobile	16.47	1.24			
	Length of stay	NHI^a^	62.25	60.55	8.89	.000	a<b,c^††^
		Medical Aid^b^	82.96	66.78			
		Automobile^c^	77.00	66.18			
Critically severe 41~75 (N = 170)	Total medical expenses	NHI	16.78	1.27	1.03	.360	
		Medical Aid	15.94	1.13			
		Automobile	16.53	1.60			
	Length of stay	NHI	60.20	66.09	1.11	.330	
		Medical Aid	45.00	82.11			
		Automobile	74.92	73.48			

^†^Bonferroni post-hoc test;

^††^Dunnett post-hoc test; NHI = National Health Insurance; ISS = Injury Severity Score; Total medical expense: converted to log value and analyzed

### Predictors of healthcare utilization among trauma patients

#### Total cost of care

We identified the factors that influenced trauma care patients’ total cost of care by performing hierarchical regression based on the Andersen behavioral model (Table 4). As the variance inflation factor (VIF) did not exceed 10, multicollinearity among independent variables was not observed. With reference to the existing literature, we entered predisposing factors first, followed by enabling factors and need factors.

**Table 4:** Factors affecting total medical expenses of trauma patients.

Variable		Model 1 (n = 7,334)					Model 2 (n = 7,286)					Model 3 (n = 7,286)					VIF
		B	SE	β	t	*p*	B	SE	β	t	*p*	B	SE	β	t	*p*	
Sex (R: Male)		.13	.03	-.05	-4.36	<.001	-.15	.03	-.06	-4.88	<.001	-.00	.03	-.00	-0.13	.893	1.051
Age		.01	.00	.22	18.64	<.001	.01	.00	.23	19.19	<.001	.01	.00	.18	18.11	<.001	1.042
Injury season (R: Spring)	Summer	-.08	.04	-.03	-1.95	.051	-.09	.04	-.04	-2.11	.035	-.11	.04	-.04	-3.05	002	1.811
	Fall	-.04	.04	-.02	-1.08	.278	-.06	.04	-.03	-1.48	.138	-.12	.04	-.05	-3.47	<.001	1.858
	Winter	-.24	.04	-.09	-5.80	<.001	-.22	.04	-.09	-5.36	<.001	-.30	.04	-.11	-8.20	<.001	1.823
ER stay (min.)							-.00	.00	-.07	-5.98	<.001	.00	.00	.03	2.75	.005	1.081
Insurance (R: NHI)	Medical Aid						.24	.07	.04	3.70	<.001	.21	.06	.04	3.64	<.001	1.024
	Automobile						.32	.03	.12	10.42	<.001	.04	.03	.01	1.34	.181	1.092
ISS												.03	.00	.28	21.82	<.001	1.690
KTAS (R: Level 2)	1											-.19	.04	-.05	-4.44	<.001	1.221
	3											-.21	.03	-.08	-6.84	<.001	1.339
	4											-.28	.04	-.09	-7.50	<.001	1.516
	5											-.34	.08	-.04	-4.20	<.001	1.100
Number of diagnosed injuries												.04	.00	.28	25.45	<.001	1.247
F						77.42***					68.54***					229.85***	
R^2^(Adj R^2^)						.05(.05)					.07(.07)					.32(.32)	
ΔR^2^(ΔAdj R^2^)											.02(.02)					.24(.24)	

*: *p* < .05;

**: *p* < .01;

***: *p* < .001;

NHI: National Health Insurance; KTAS: Korean Triage and Acuity Scale; ISS: Injury Severity Score; ^†^ED stay: Emergency department stay, including missing data (non-missing = 7286)

In model 1, we entered sex, age, and season of trauma. Although all the predisposing factors did not have high explanatory power (Adj R^2^ = .052, *p* < .001), sex, age, and winter (season of trauma) had a significant influence on total cost of care (F = 77.42, *p* < .001).

In model 2, we added the enabling factors of duration of ED stay and insurance type per model 1. Model 2 was fitted (F = 68.54, *p* < .001), with a change of Adj R^2^ of .020. The predisposing factors of sex (β = -.06, *p* < .001), age (β = .23, *p* < .001) and winter (β = -.09, *p* < .001) had significant effects on total cost of care for trauma patients. Total cost of care decreased with increasing duration of ED stay, the enabling factor (β = -.07, *p* < .001). Total cost of care was higher with medical aid claims (β = .04, *p* < .001) and automobile insurance claims (β = .12, *p* < .001) compared to NHI claims.

In model 3, we added need factors, which were ISS, KTAS, and number of injury codes per model 2. Model 3 was also fitted (F = 229.85, *p* < .001), with a change of Adj R^2^ of .243. Model 3 explained the most variance in total cost of care and confirmed that need factors had the greatest impact on total cost of care. The predisposing factors of age (β = .18, *p* < .001), season of trauma (summer β = -.04, *p* = .002; fall β = -.05, *p* < .001; winter β = -.11, *p* < .001), duration of ED stay (β = .03, *p* = .005), and insurance type (medical aid β = .04, *p* < .001) were significantly associated with total cost of care. In terms of need factors, total cost of care increased with increasing ISS (β = .28, *p* < .001), and total cost of care was lower with KTAS level 1 (β = -.05, *p* < .001), level 3 (β = -.08, *p* < .001), level 4 (β = -.09, *p* < .001), and level 5 (β = -.04, *p* < .001) compared to level 2. Furthermore, the total cost of care increased with an increasing number of injury codes (β = .28, *p* < .001). The number of injury codes had the greatest effect on trauma patients’ total cost of care, followed by ISS and age.

#### Length of hospital stay in days

Table 5 shows the results of the negative binomial regression, performed to estimate the factors that impact trauma patients’ length of hospital stay. A deviance/df, which represents the degree of deviance of data from the negative binomial regression, closer to 1 indicates that the data is suitable for negative binomial analysis, and a deviance/df greater than 4 indicates that the model is not fitted [26]. Total deviance/df in our study was 1.169, which confirmed that the data was suitable for negative binomial analysis. The results indicate that the length of hospital stay would show a 13% increase with women than with men (OR 1.13, 95% CI 1.07–1.09, *p* < .001), a 1% increase with each additional year of age (OR 1.01, 95% CI 1.01–1.01, *p* < .001), and an 8% decrease when trauma occurred in the summer compared to spring (OR 0.92, 95% CI 0.85–0.99, *p* = .019). Length of hospital stay would show a 62% increase with automobile insurance (OR 1.62, 95% CI 1.54–1.71, *p* < .001) and a 53% increase with medical aid (OR 1.53, 95% CI 1.36–1.72, *p* < .001) compared to NHI. The length of hospital stay would show a 2% increase with each point increase in the ISS (OR 1.02, 95% CI 1.02–1.03, *p* < .001), and a 42% increase for KTAS level 2 (OR 1.42, 95% CI 1.30–1.54, *p* < .001), a 26% increase for level 3 (OR 1.26, 95% CI 1.14–1.39, *p* < .001), a 24% increase for level 4 (OR 1.24, 95% CI 1.11–1.38, *p* < .001), and a 27% increase in level 5 (OR 1.27, 95% CI 1.06–1.52, *p* = .010) compared to level 1. The length of hospital stay would show a 3% increase with each additional diagnosed injury code (OR 1.03, 95% CI 1.03–1.04, *p* < .001).

**Table 5:** Factors affecting length of stay in trauma patients (*N* = 7,334).

Variable	Class	B	SE	OR (95% CI)	*p*
Sex (R: Male)	Female	.12	.03	1.13 (1.07–1.19)	<.001
Age		.01	.00	1.01 (1.01–1.01)	<.001
Injury season (R: Spring)	Winter	-.05	.04	0.95 (0.88–1.02)	.156
	Fall	-.07	.04	0.93 (0.87–1.00)	.050
	Summer	-.09	.04	0.92 (0.85–0.99)	.019
	Spring	.00	.00	1.00	.
^†^ED stay		.00	.00	1.00 (1.00–1.00)	<.001
Insurance (R: NHI)	Automobile	.48	.03	1.62 (1.54–1.71)	<.001
	Medical Aid	.42	.06	1.53 (1.36–1.72)	<.001
	NHI	.00	.00	1.00	.
ISS		.02	.00	1.02 (1.02–1.03)	<.001
KTAS (R: Level 1)	5	.24	.09	1.27 (1.06–1.52)	.010
	4	.21	.06	1.24 (1.11–1.38)	<.001
	3	.23	.05	1.26 (1.14–1.39)	<.001
	2	.35	.04	1.42 (1.30–1.54)	<.001
	1	.00	.00	1.00	.
Number of diagnosed injuries		.03	.00	1.03 (1.03–1.04)	<.001
deviance/df = 1.17					

OR: odds ratio; NHI: National Health Insurance; KTAS: Korean Triage and Acuity Scale; ISS: Injury Severity Score;

^†^ED stay: Emergency department stay, including missing data (non-missing = 7,286)

## Discussion

This study compared and analyzed trauma patients’ healthcare utilization by insurance type and the ISS and identified the predictors of healthcare utilization based on the Andersen behavioral model. We analyzed healthcare big data of trauma cases in 2016, published by the HIRA, and the results showed that trauma patients’ healthcare utilization is determined by injury severity. Total cost of care was most influenced by need factors, suggesting equality in healthcare utilization, while length of hospital stay in days differed markedly by insurance type, which suggests that health services are utilized by personal choice.

The findings of this study were consistent with the 2016 United States annual report [9] that the average length of hospital stay, in days, increases with an increasing ISS severity. Although it may seem natural that total cost of care and length of hospital stay increase with increasing injury severity, our results, which were obtained by analyzing total cost of care and length of hospital stay for six months according to the ISS, have significant implications. In particular, total cost of care did not significantly differ according to the ISS among patients that had a very severe injury with an ISS of 25 or higher, and length of hospital stay did not significantly differ among patients with a severe injury with an ISS of 16 or higher. This shows that there are no differences in healthcare utilization, as determined by total cost of care and length of hospital stay, among patients with a severe injury. This suggests that it is imperative that patients diagnosed with a severe or more critical injury be sufficiently treated to resolve their ongoing health problems and minimize consequent disabilities, regardless of an additional diagnosis of injury severity.

To accurately compare healthcare utilization by insurance type, we analyzed the variations of healthcare utilization according to insurance type after stratifying the ISS. Results showed that among patients with mild or moderate injury with an ISS of 15 or lower, total cost of care was the highest for medical aid claims while length of hospital stay was the highest for automobile insurance claims, followed by medical aids and NHI claims. This is speculated to be because those with lower financial status have difficulty accessing care, which makes them more vulnerable to worsening of their illness compared to those with the same disease severity but have NHI [27]. The fact that the length of hospital stay was greatest with automobile insurance claims, among patients with low injury severity, is likely attributable to healthcare utilization beyond need among patients with mild conditions, as suggested by a previous study [7]. This highlights the need for a reasonable system to shorten the length of hospital stays with automobile insurance claims cases involving minor injuries. However, the length of hospital stay did not differ according to insurance type among patients with critical injuries, with an ISS of 41 or higher. Therefore, it is necessary to revise the medical care fee system for trauma care patients, such as by expanding the coverage of treatments that are proven to be essential and effective, so that severe trauma patients can utilize health services sufficiently.

Regarding the predictors for total cost of care for trauma patients, age was confirmed as a significant predictor for total cost of care in models 1, 2, and 3. As age is a predictor and an important risk factor for mortality in elderly trauma patients [28], and older adults are more likely to incur severe trauma than younger counterparts [29], total cost of care is predicted to increase with advancing age. Particularly, middle-aged adults in their 50s or older accounted for 60% of our participants. Hence, in addition to implementing systems to lower the incidence of trauma among older adults, it would be important to explore measures for aggressive treatment and management at the early stages of trauma.

Insurance type, which was found to have a strong influence in model 2, had a lower influence in model 3 after adding need factors. This shows that the influence of insurance type on total cost of care diminished as that of need factors increased, suggesting that total cost of care is determined by injury severity. Hence, instead of simply developing policies or measures pertaining to reducing total cost of care based on insurance type, there should be a discussion about policies for lowering total cost of care by reducing the gap in health service provisions among insurance types for patients with severe injuries, considering injury severity in addition to insurance type.

Among the need factors, the ISS was identified as a major factor in total cost of care. Total cost of care increased with increasing ISS, which was consistent with the findings of Garnett and Browne [11], and Kuwabara et al. [30]. This again confirmed that the ISS enables the prediction of necessary resources for trauma patient management [9] and is a major predictor of healthcare utilization in trauma patients [9,11–13]. Therefore, analysis and interpretation based on the ISS is expected to produce more reliable results pertaining to trauma patients’ healthcare utilization, particularly the total cost of care.

The number of injury codes was also a major factor in total cost of care, the latter increasing with the former, in line with the results of Lee and Yom [31]. Particularly, the injury codes add information about injuries that are excluded in the ISS calculation. The ISS is computed by dividing the body into six parts to calculate the Abbreviated Injury Scale (AIS) score for each part and adding the squares of the top three AIS scores [32]. Thus, subsequent studies that analyze healthcare utilization among trauma patients should also include the number of injury codes as a major variable. In addition, comorbidity is reported as an important co-factor to explain the use of health services [33]. However, we analyzed the number of injury diagnoses as a supplement to comorbidity. In a future study, we can consider comorbidity as an important factor through the analysis of additional information such as the Charlson Comorbidity Index [34].

The results confirm that the need factors of the Andersen model are the major factors in total cost of care in Korean trauma patients, suggesting that there is equity in healthcare utilization. This concurs with the results of Lee and Yom [31] and Jourdan et al. [35] and implies that trauma patients’ healthcare utilization is determined by the severity of the injury.

Length of hospital stay, another parameter of trauma patients’ healthcare utilization, is an important index that influences patients’ cost of care and convenience, and thus needs efficient management [36]. Our results show that medical aid recipients had longer hospital stays than those with NHI. Health status worsens with decreasing income level [37], and medical aid recipients stay in the hospital for longer [38]. Further, total cost of care did not significantly differ between automobile insurance claims and NHI claims, but length of hospital stay was longer with automobile insurance claims. This is presumably because automobile insurance claims have no out-of-pocket costs for the patient and long hospital stays are advantageous when negotiating compensation with the insurance company. According to Shin et al. [7], length of hospital stay with automobile insurance claims is shorter or commensurate with NHI claims in tertiary hospitals but longer in hospitals or clinics. Hence, subsequent studies should also perform an analysis for different types of healthcare facilities.

Regarding the effect of KTAS, length of hospital stay was longer among patients at KTAS levels 2, 3, 4, and 5 compared to those at KTAS level 1, which is a life-threatening, critical status. However, patients with KTAS level 5 (non-urgent) had longer hospital stays during the six months after the onset of trauma compared to patients at levels 3 and 4 (urgent and less urgent, respectively). As this can increase the cost of national healthcare, relevant measures should be developed by identifying the exact causes of lengthy hospital stays among patients at KTAS level 5 compared to those at KTAS levels 3 and 4 to ensure appropriate hospital bed utilization. Thus, it is essential to develop measures to regulate the length of hospital stay for patients with mild and moderate injuries, which was the group of patients whose length of hospital stay differed by insurance type.

## Limitations

Drawing from healthcare big data published by the HIRA, this study analyzed the claims data containing KTAS and the ISS for patients who incurred trauma in 2016. Hence, we speculate that our participants were trauma patients who were admitted to regional level I trauma centers in Korea, meaning our sample is limited in representation of the entire trauma population in Korea. Further, our data were written by hospitals to claim reimbursement for healthcare costs, and the diagnosis codes may be inaccurate. In addition, there was a substantial gap in the number of NHI, medical aid, and automobile insurance claims in our data. However, we used big data in Korea that represented the total population and tried to normalize the data with statistical methods. Therefore, these limitations should be considered when interpreting and generalizing the results of this study.

## Conclusion

This study identified the predictors of healthcare utilization among trauma patients in Korea based on the Andersen behavioral model for healthcare utilization using the 2016 HIRA trauma patient data. The total cost of care in Korean trauma patients was found to be heavily influenced by need factors, confirming that there is equity in healthcare utilization. However, length of hospital stay in days differed according to insurance type, suggesting that personal choice impacts healthcare utilization. Our results confirmed that the severity of trauma determines patients’ healthcare utilization, revealing the need to expand insurance coverage for severe trauma patients, implement practical revisions to the medical fee system, and strengthen the review of patients with mild and moderate injuries to promote more effective and efficient healthcare utilization. Subsequent studies should employ various types of big data to analyze the factors of healthcare utilization from multiple aspects proposed by the Andersen model.

## Supporting information

S1 Appendix Flowchart of selection of study subjects. KTAS: Korean Triage and Acuity Scale; ISS: Injury Severity Score; NHI: National Health Insurance.
(PDF)

S2 Appendix Health Insurance Review & Assessment Service (HIRA) healthcare big data remote analysis service process. HIRA: Health Insurance Review & Assessment Service; PC: Personal computer.
(PDF)

S1 Table General characteristics of the participants (*N* = 7,334). ^†^ED stay: Emergency department stay, including missing data (non-missing = 7286); NHI: National Health Insurance; KTAS: Korean Triage and Acuity Scale; ISS: Injury Severity Score.
(PDF)

S2 Table Health service use according to insurance type and ISS (*N* = 7,334). ^†^Bonferroni post-hoc test; ^††^Dunnett post-hoc test; Total medical expense: converted to log value and analyzed; NHI = National Health Insurance; ISS = Injury Severity Score.
(PDF)

S3 Table Health service use by insurance type according to ISS. ^†^Bonferroni post-hoc test; ^††^Dunnett post-hoc test; NHI = National Health Insurance; ISS = Injury Severity Score; Total medical expense: converted to log value and analyzed.
(PDF)

S4 Table Factors affecting total medical expenses of trauma patients. *: *p* < .05; **: *p* < .01; ***: *p* < .001; NHI: National Health Insurance; KTAS: Korean Triage and Acuity Scale; ISS: Injury Severity Score; ^†^ED stay: Emergency department stay, including missing data (non-missing = 7286).
(PDF)

S5 Table Factors affecting length of stay in trauma patients (*N* = 7,334). OR: odds ratio; NHI: National Health Insurance; KTAS: Korean Triage and Acuity Scale; ISS: Injury Severity Score; ^†^ED stay: Emergency department stay, including missing data (non-missing = 7,286).
(PDF)
