# Supplementary figures and images for: Factors influencing the use of health services by trauma patients according to insurance type and injury severity score in South Korea: Based on Andersen’s behavioral model
Source: PLoS One. 2020 Aug 27;15(8):e0238258. doi: 10.1371/journal.pone.0238258 (PMC7451573; doi:10.1371/journal.pone.0238258)

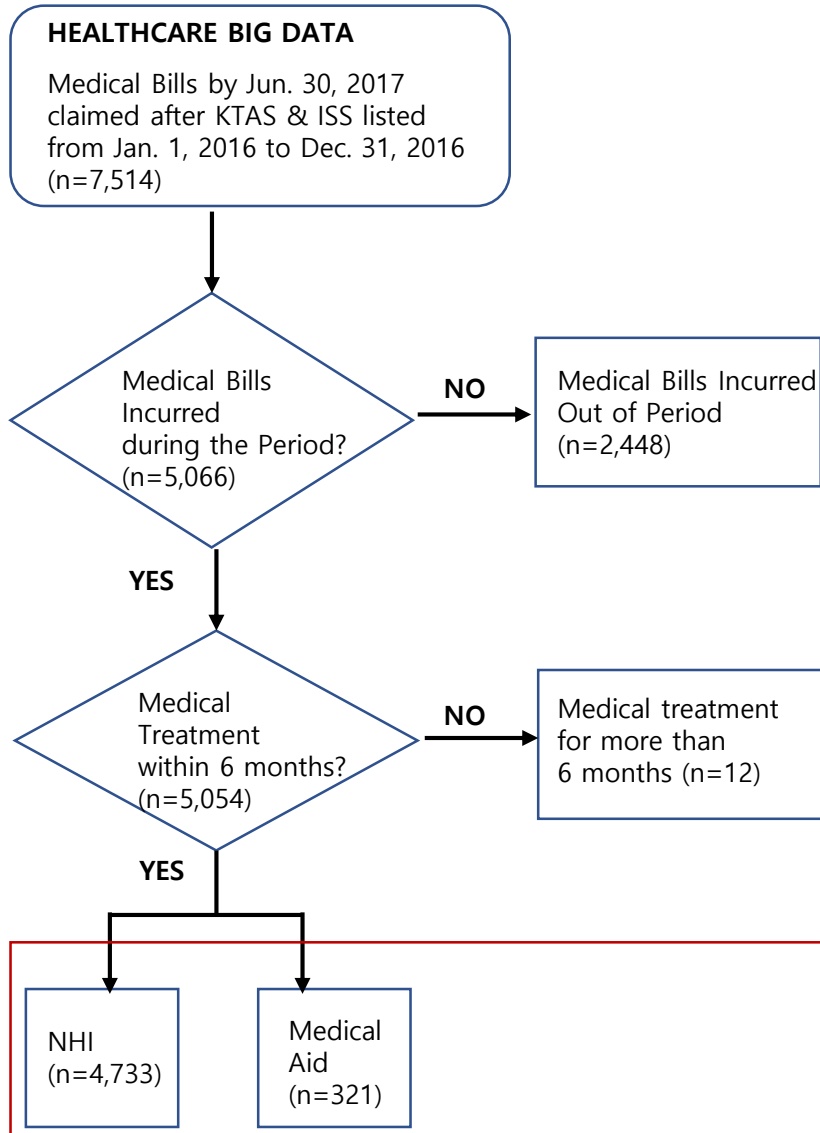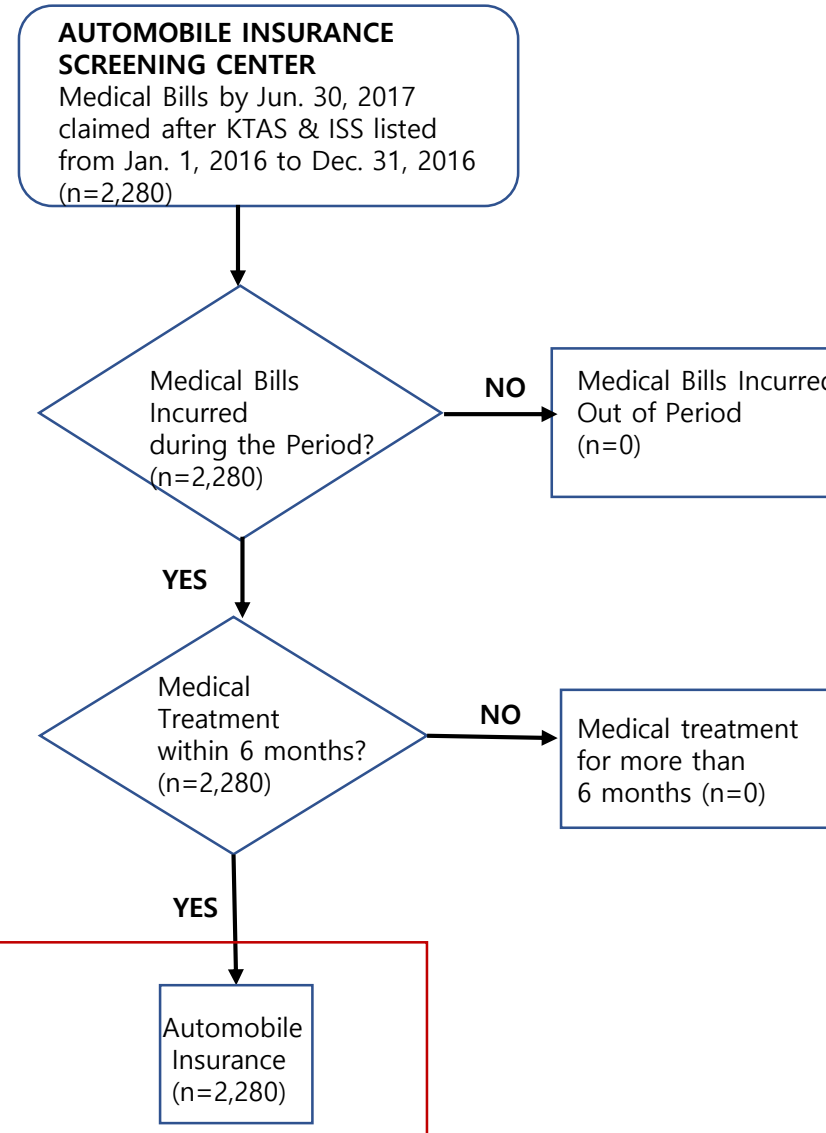

Supplement: S1 Appendix — KTAS: Korean Triage and Acuity Scale; ISS: Injury Severity Score; NHI: National Health Insurance. (PDF) [file pone.0238258.s001.pdf]

## RESEARCHER

## HIRA

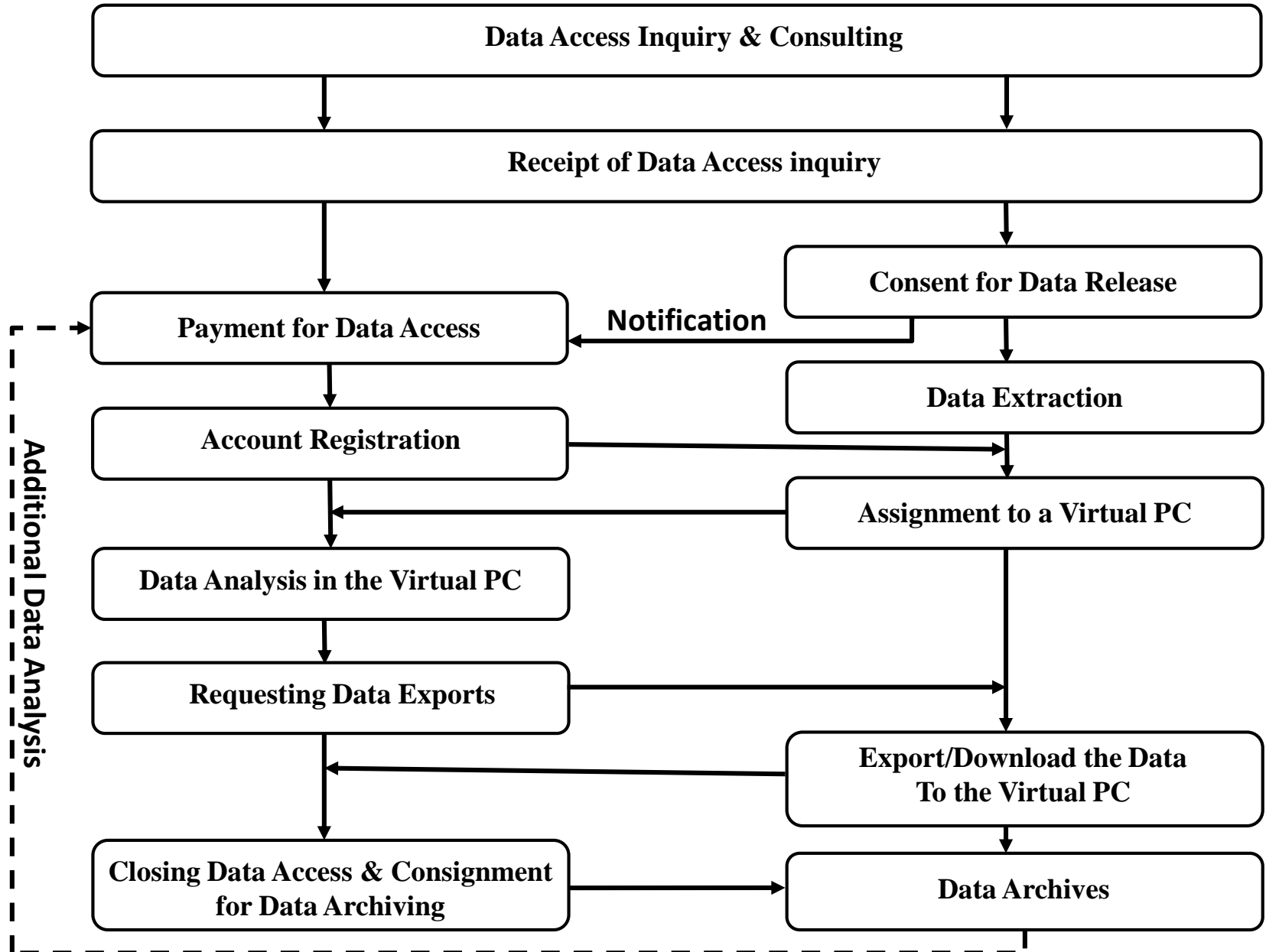

Supplement: S2 Appendix — HIRA: Health Insurance Review & Assessment Service; PC: Personal computer. (PDF) [file pone.0238258.s002.pdf]
