# Supplementary material for: Factors influencing the use of health services by trauma patients according to insurance type and injury severity score in South Korea: Based on Andersen’s behavioral model
Source: PLoS One. 2020 Aug 27;15(8):e0238258. doi: 10.1371/journal.pone.0238258 (PMC7451573; doi:10.1371/journal.pone.0238258)
Supplement: S1 Table — †ED stay: Emergency department stay, including missing data (non-missing = 7286); NHI: National Health Insurance; KTAS: Korean Triage and Acuity Scale; ISS: Injury Severity Score. (PDF) [file pone.0238258.s003.pdf]

Characteristics	Class	NHI n (%)	Medical aid n (%)	Automobile n (%)	Total n (%)
**Sex**	Male	3385(71.5)	219(68.2)	1,508(66.1)	5,112(69.7)
	Female	1,348(28.5)	102(31.8)	772(33.9)	2,222(30.3)
**Age (yrs)**	0~9	249(5.3)	7(2.2)	69(3.0)	325(4.4)
	10~19	247(5.2)	11(3.4)	126(5.5)	384(5.2)
	20~29	403(8.5)	15(4.7)	224(9.8)	642(8.8)
	30~39	379(8.0)	15(4.7)	223(9.8)	617(8.4)
	40~49	567(12.0)	32(9.9)	314(13.8)	913(12.5)
	50~59	902(19.0)	92(28.7)	393(17.2)	1,387(18.9)
	60~69	781(16.5)	57(17.7)	414(18.2)	1,252(17.1)
	70~79	719(15.2)	58(18.1)	371(16.3)	1,148(15.6)
	≥80	486(10.3)	34(10.6)	146(6.4)	666(9.1)
**Season of trauma**	Spring	857(18.1)	62(19.3)	374(16.4)	1,293(17.6)
	Summer	1,243(26.3)	78(24.3)	561(24.6)	1,882(25.7)
	Fall	1,306(27.6)	87(27.1)	660(29.0)	2,053(28.0)
	Winter	1,327(28.0)	94(29.3)	685(30.0)	2,106(28.7)
**ED stay (min)**[†]		266.98±265.58	306.51±351.53	245.80±267.25	262.09±270.71
**KTAS**	1 Resuscitation	383(8.1)	38(11.8)	333(14.6)	754(10.3)
	2 Emergency	1,861(39.3)	135(42.1)	1,227(53.8)	3,223(43.9)
	3 Urgency	1,288(27.2)	73(22.7)	502(22.1)	1,863(25.4)
	4 Less Urgency	1,069(22.6)	65(20.3)	100(8.3)	1,324(18.1)
	5 Nonurgency	132(2.8)	10(3.1)	28(1.2)	170(2.3)
**ISS**	Mild (1~8)	1,880(39.7)	106(33.0)	575(25.2)	2,561(34.9)
	Moderate (9~15)	1,012(21.4)	80(24.9)	514(22.6)	1,606(21.9)

	Severe (16~24)	1,030(21.7)	74(23.0)	650(28.5)	1,754(23.9)
	Very severe (25~40)	732(15.5)	57(17.8)	454(19.9)	1,243(17.0)
	Critically severe (41~75)	79(1.7)	4(1.3)	87(3.8)	170(2.3)
**Number of diagnosed injuries**		7.08±8.85	6.97±12.33	10.40±8.95	8.11±9.19
**Survival**	Alive	4,541(95.9)	308(95.9)	2,076(91.1)	6,925(94.4)
	Death	192(4.1)	13(4.1)	204(8.9)	409(5.6)
**Total medical expense (1,000 won)**		12,390±16.37	146,27±15,70	13,599±16,56	12,864±16,41
**Length of stay (days)**		28.61±38.93	47.28±53.39	55.20±48.93	49.57±53.96
