# Supplementary material for: Factors influencing the use of health services by trauma patients according to insurance type and injury severity score in South Korea: Based on Andersen’s behavioral model
Source: PLoS One. 2020 Aug 27;15(8):e0238258. doi: 10.1371/journal.pone.0238258 (PMC7451573; doi:10.1371/journal.pone.0238258)
Supplement: S2 Table — †Bonferroni post-hoc test; ††Dunnett post-hoc test; Total medical expense: converted to log value and analyzed; NHI = National Health Insurance; ISS = Injury Severity Score. (PDF) [file pone.0238258.s004.pdf]

Variable	Class		Mean	SD	F	*p*	Post-hoc
Total medical expense	Insurance Type	NHI[a]	15.70	1.16	55.80	<.001	a<b,c†
		Medical Aid[b]	15.98	1.11			
		Automobile[c]	16.02	1.19			
	ISS	1~8[a]	15.10	0.90	534.54	<.001	a<b<c<d,e††
		9~15[b]	15.81	1.00			
		16~24[c]	16.25	1.10			
		25~40[d]	16.52	1.16			
		41~75[e]	16.64	1.43			
Length of stay	Insurance Type	NHI[a]	39.81	48.67	212.62	<.001	a<b<c††
		Medical Aid[b]	60.03	59.77			
		Automobile[c]	68.36	58.15			
	ISS	1~8[a]	24.94	32.98	321.24	<.001	a<b<c,d,e††
		9~15[b]	54.67	52.58			
		16~24[c]	65.68	57.28			
		25~40[d]	67.38	63.37			
		41~75[e]	68.59	70.34			
