# Supplementary material for: Factors influencing the use of health services by trauma patients according to insurance type and injury severity score in South Korea: Based on Andersen’s behavioral model
Source: PLoS One. 2020 Aug 27;15(8):e0238258. doi: 10.1371/journal.pone.0238258 (PMC7451573; doi:10.1371/journal.pone.0238258)
Supplement: S3 Table — †Bonferroni post-hoc test; ††Dunnett post-hoc test; NHI = National Health Insurance; ISS = Injury Severity Score; Total medical expense: converted to log value and analyzed. (PDF) [file pone.0238258.s005.pdf]

ISS	Variable	Class	Mean	SD	F	*p*	Post-hoc
Mild (1~8) (n=2,561)	Total medical expenses	NHI[a]	15.02	0.85	29.26	<.001	a,c<b[††]
		Medical Aid[b]	15.40	0.88			
		Automobile[c]	15.36	1.01			
	Length of stay	NHI[a]	19.34	26.60	74.99	<.001	a<b<c[††]
		Medical Aid[b]	33.64	44.15			
		Automobile[c]	41.62	42.21			
Moderate (9~15) (n=1,606)	Total medical expenses	NHI[a]	15.73	1.01	10.43	<.001	a<c<b[†]
		Medical Aid[b]	16.05	0.91			
		Automobile[c]	15.95	0.96			
	Length of stay	NHI[a]	45.85	50.61	40.79	<.001	a<b,c[†]
		Medical Aid[b]	65.36	59.19			
		Automobile[c]	70.39	51.43			
Severe (16~24) (n=1,754)	Total medical expenses	NHI	16.23	1.06	0.51	.600	
		Medical Aid	16.24	1.25			
		Automobile	16.29	1.15			
	Length of stay	NHI[a]	53.74	52.57	55.90	<.001	a<b,c[††]
		Medical Aid[b]	75.20	60.58			
		Automobile[c]	83.50	59.25			
Very severe 25~40 (N=1,243)	Total medical expenses	NHI	16.55	1.13	1.01	.365	
		Medical Aid	16.66	1.04			
		Automobile	16.47	1.24			
	Length of stay	NHI[a]	62.25	60.55	8.89	.000	a<b,c[††]
		Medical Aid[b]	82.96	66.78			
		Automobile[c]	77.00	66.18			
Critically severe 41~75 (N=170)	Total medical expenses	NHI	16.78	1.27	1.03	.360	
		Medical Aid	15.94	1.13			
		Automobile	16.53	1.60			
	Length of stay	NHI	60.20	66.09	1.11	.330	
		Medical Aid	45.00	82.11			
		Automobile	74.92	73.48			
