# Supplementary material for: Factors influencing the use of health services by trauma patients according to insurance type and injury severity score in South Korea: Based on Andersen’s behavioral model
Source: PLoS One. 2020 Aug 27;15(8):e0238258. doi: 10.1371/journal.pone.0238258 (PMC7451573; doi:10.1371/journal.pone.0238258)
Supplement: S4 Table — *: p < .05; **: p < .01; ***: p < .001; NHI: National Health Insurance; KTAS: Korean Triage and Acuity Scale; ISS: Injury Severity Score; †ED stay: Emergency department stay, including missing data (non-missing = 7286). (PDF) [file pone.0238258.s006.pdf]

Variable		Model 1 (n = 7,334)					Model 2 (n = 7,286)					Model 3 (n = 7,286)					VIF
		B	SE	β	t	*p*	B	SE	β	t	*p*	B	SE	β	t	*p*	
Sex (R: Male)		.13	.03	-.05	-4.36	<.001	-.15	.03	-.06	-4.88	<.001	-.00	.03	-.00	-0.13	.893	1.051
Age		.01	.00	.22	18.64	<.001	.01	.00	.23	19.19	<.001	.01	.00	.18	18.11	<.001	1.042
Injury season (R: Spring)	Summer	-.08	.04	-.03	-1.95	.051	-.09	.04	-.04	-2.11	.035	-.11	.04	-.04	-3.05	002	1.811
	Fall	-.04	.04	-.02	-1.08	.278	-.06	.04	-.03	-1.48	.138	-.12	.04	-.05	-3.47	<.001	1.858
	Winter	-.24	.04	-.09	-5.80	<.001	-.22	.04	-.09	-5.36	<.001	-.30	.04	-.11	-8.20	<.001	1.823
ER stay (min.)							-.00	.00	-.07	-5.98	<.001	.00	.00	.03	2.75	.005	1.081
Insurance (R: NHI)	Medical Aid						.24	.07	.04	3.70	<.001	.21	.06	.04	3.64	<.001	1.024
	Automobile						.32	.03	.12	10.42	<.001	.04	.03	.01	1.34	.181	1.092
ISS												.03	.00	.28	21.82	<.001	1.690
KTAS (R: Level 2)	1											-.19	.04	-.05	-4.44	<.001	1.221
	3											-.21	.03	-.08	-6.84	<.001	1.339
	4											-.28	.04	-.09	-7.50	<.001	1.516
	5											-.34	.08	-.04	-4.20	<.001	1.100
Number of diagnosed injuries												.04	.00	.28	25.45	<.001	1.247
F						77.42^{***}					68.54^{***}					229.85^{***}	
R^2(Adj R^2)						.05(.05)					.07(.07)					.32(.32)	

△R^2(△Adj R^2)										.02(.02)					.24(.24)	
