# Supplementary material for: Factors influencing the use of health services by trauma patients according to insurance type and injury severity score in South Korea: Based on Andersen’s behavioral model
Source: PLoS One. 2020 Aug 27;15(8):e0238258. doi: 10.1371/journal.pone.0238258 (PMC7451573; doi:10.1371/journal.pone.0238258)
Supplement: S5 Table — OR: odds ratio; NHI: National Health Insurance; KTAS: Korean Triage and Acuity Scale; ISS: Injury Severity Score; †ED stay: Emergency department stay, including missing data (non-missing = 7,286). (PDF) [file pone.0238258.s007.pdf]

Variable	Class	B	SE	OR (95% CI)	*p*
Sex (R: Male)	Female	.12	.03	1.13 (1.07-1.19)	<.001
Age		.01	.00	1.01 (1.01-1.01)	<.001
Injury season (R: Spring)	Winter	-.05	.04	0.95 (0.88-1.02)	.156
	Fall	-.07	.04	0.93 (0.87-1.00)	.050
	Summer	-.09	.04	0.92 (0.85-0.99)	.019
	Spring	.00	.00	1.00	.
†ED stay		.00	.00	1.00 (1.00-1.00)	<.001
Insurance (R: NHI)	Automobile	.48	.03	1.62 (1.54-1.71)	<.001
	Medical Aid	.42	.06	1.53 (1.36-1.72)	<.001
	NHI	.00	.00	1.00	.
ISS		.02	.00	1.02 (1.02-1.03)	<.001
KTAS (R: Level 1)	5	.24	.09	1.27 (1.06-1.52)	.010
	4	.21	.06	1.24 (1.11-1.38)	<.001
	3	.23	.05	1.26 (1.14-1.39)	<.001
	2	.35	.04	1.42 (1.30-1.54)	<.001
	1	.00	.00	1.00	.
Number of diagnosed injuries		.03	.00	1.03 (1.03-1.04)	<.001
deviance/df=1.17					
